# Barriers to advance care planning: a qualitative study of seriously ill Chinese patients and their families

**DOI:** 10.1186/s12904-020-00587-0

**Published:** 2020-06-08

**Authors:** Johnny T. K. Cheung, Doreen Au, Anthony H. F. Ip, Jenny Chan, Kenway Ng, Lok Cheung, Jacqueline Yuen, Elsie Hui, Jenny Lee, Raymond Lo, Jean Woo

**Affiliations:** 1 Institute of Ageing, The Chinese University of Hong Kong, Hong Kong SAR, China; 2 Department of Medicine & Geriatrics, Shatin Hospital, Hong Kong SAR, China; 3 Department of Medicine & Geriatrics, Tai Po Hospital, Hong Kong SAR, China; 4 Department of Medicine & Therapeutics, Faculty of Medicine, The Chinese University of Hong Kong, Hong Kong SAR, China

**Keywords:** End of life care, Palliative care, Advance directives, Do-not-attempt cardiopulmonary resuscitation

## Abstract

**Background:**

Advance care planning (ACP) facilitates identification and documentation of patients’ treatment preferences. Its goal aligns with that of palliative care – optimizing quality of life of seriously ill patients. However, concepts of ACP and palliative care remain poorly recognized in Chinese population. This study aims at exploring barriers to ACP from perspective of seriously ill patients and their family caregivers.

**Methods:**

This is a qualitative study conducted in a Palliative Day Care Centre of Hong Kong between October 2016 and July 2017. We carried out focus groups and individual interviews for the seriously ill patients and their family caregivers. A semi-structured interview guide was used to explore participants’ experiences and attitudes about ACP. Qualitative content analysis was adopted to analyze both manifest content and latent content.

**Results:**

A total of 17 patients and 13 family caregivers participated in our study. The qualitative analysis identified four barriers to ACP: 1) limited patients’ participation in autonomous decision making, 2) cognitive and emotional barriers to discussion, 3) lack of readiness and awareness of early discussion, and 4) unprepared healthcare professionals and healthcare system*.*

**Conclusions:**

Participations of seriously ill patients, family caregivers and healthcare workers in ACP initiation are lacking respectively. A series of interventions are necessary to resolve the barriers.

## Background

Advance care planning (ACP) is an overarching process of proactive communication regarding end-of life care [1]. Patients with advanced progressive disease make decisions regarding future medical care aligned with their individual preferences, in advance of mental incapability of decision making. Specifically, ACP for older adults includes several elements: 1) discussions about values and preferences, 2) discussions with and designation of surrogate decision makers, 3) discussions about treatment options, 4) advance directives (AD) documentation [2]. Early and ongoing ACP conversations between patients, families, and healthcare professionals can prepare patients for discussing specific goals of care [3].

Palliative care improves quality of life of seriously ill patients and their family members through early prevention and relief of suffering [4]. Palliative care units have been recognized as appropriate setting to implement ACP [5]. ACP facilitates identification and documentation of individual preferences. This aligns with goal of palliative care – optimizing quality of life of seriously ill patients [6]. Additional benefits of ACP include reduced hospitalization, unnecessary futile treatments and cost, and stress, anxiety and depression of families [2].

Meanwhile, in Hong Kong, palliative care and ACP are poorly recognized. Given that over 90% of deaths occur in hospitals [7] and the fact that Hong Kong population enjoys a high life expectancy at 60 [8], there will be a growing demand for palliative care in Hong Kong. Nonetheless, Hong Kong was ranked relatively low in terms of palliative and healthcare environment and community engagement, lagging behind many other developed regions [9]. For instance, the Patient Self-Determination Act in the United State requires most health care institutions to inform patients of their right to refuse medical care and right to provide advance direction [10]. The legislation successfully motivates majority of community-dwelling older Americans to complete AD [11, 12]. These patients received care that was strongly associated with their preferences, according to the data from the Health and Retirement Study [13]. In comparison, there is currently no legislation establishing the legal status of AD in Hong Kong or in the rest of the China. As a result, majority of the citizens had not heard about AD or did not prefer its documentation, although they were willing to discuss end-of-life issues and were unwilling to endure life-sustaining interventions [14, 15]. A local clinical chart review shows that more than half of ACP discussions were first documented within a month before death, despite early presentations of illnesses [16]. Unfamiliarly or novelty of the concept of ACP are suggested as major barriers to its initiation in Chinese community [15].

Recognizing the delay in ACP uptake, implementing interventions in community and healthcare settings is necessary. This requires an understanding of existing barriers hindering ACP uptake. Nevertheless, previous quantitative and qualitative studies were mostly conducted on healthcare professionals [17–21]. For example, a Canadian clinician survey reveals that family member-related and patient-related factors are the most important barriers to goals of care discussions, whereas clinicians perceive their own skills and system factors as less important barriers [19]. However, there is limited evidence reflecting perspectives of patients and family caregivers, who are key participants of ACP. They may have different attitudes and perspectives. Additionally, most of the studies exploring barriers to ACP are conducted in Western countries [18]. Despite being a former British colony, Hong Kong society is still highly influenced by traditional Chinese cultural values. Such a culture may influence citizens’ attitude and perspective. In view of the significance and dearth of research, this study aims to explore barriers to ACP among seriously ill patients and their family members in Hong Kong.

## Methods

### Study background

This study is a part of Jockey Club End-of-life Care Community Project, which implements a capacity building and education programme in hospitals, residential care homes for the elderly, and the community at the New Territories East Cluster of the Hospital Authority in Hong Kong [22]. The programme covered a wide variety of topics including communication skills in serious illness conversations and symptom management in end-of-life care through talks, workshops, forums, seminars, and conferences. It aims to improve the end-of-life care practices in hospitals through healthcare staff empowerment.

### Study design and setting

This study adopted a qualitative approach to explore terminally ill patients’ and their family members’ attitude towards and understanding of discussion on end-of-life issue. It was conducted in a palliative day care centre of a rehabilitative hospital in Hong Kong, where a multidisciplinary team cared for cancer and end-stage non-cancer patients. The centre aimed to maximize patients’ quality of life through symptom control, rehabilitative activities, psychological and social support, and recreational activities.

### Subjects and procedures

Eligible subjects were palliative day care patients and their family caregivers aged 18 or above. They had no ACP initiation and documentation. Those with obvious cognitive and language impairment or unable to understand and speak Cantonese were excluded. We used purposive sampling to recruit the eligible subjects through referrals by nurses working in the centre. Subjects were sampled until content saturation was reached. We adopted the model of inductive thematic saturation, which suggests the extent to which new codes or themes are identified within the data, rather than the completeness of existing categories [23]. In other words, we considered that further data collection would not generate new insights once no additional meaningful units generated.

We carried out focus group discussion to explore their complex personal experiences, beliefs, perceptions and attitudes through moderated interaction [24]. After conducting three focus groups of patients to discuss, we found that a number of participants were unable to meet at a particular time together. Therefore, we conducted individual in-depth interviews instead. Similar to focus group discussion, interviews can also uncover informants’ perceptions and attitudes [24].

### Data collection

Focus group discussions and individual interviews were conducted in a private room inside the palliative day care centre between November 2016 and July 2017. Two moderators asked questions and led the discussions or interviews. In order to allow flexibility, we used a semi-structured interview guide to explore participants’ experience in and attitude towards three subtopics: 1) goals of care communication, 2) information seeking and understanding, and 3) end-of-life decision making. The interview guide was developed based on discussions with experienced researchers and workers in end of life care (Additional file 1 and 2). For example, we asked the patients and their family members their motivation for knowing medical conditions and their actual information seeking behaviour (question 2 and 3), in order to explore their willingness to discuss end-of-life issues and attitude-behaviour consistency. Supplementary and probing questions were also asked to clarify responses and to pursue new ideas. The individual interviews and the focus groups lasted for 20–30 min and approximately 1 h respectively. All interviews were audio-taped for accurate data transcription.

Prior to the interviews, a written informed consent was obtained from every participant. Ethical approval was granted from The Joint Chinese University of Hong Kong – New Territories East Cluster Clinical Research Ethics Committee.

### Data analysis

A nursing student transcribed the audio-recorded interviews verbatim in Cantonese. We then analyzed the transcripts using qualitative content analysis, which focused on both manifest and latent content [25]. Manifest content refers to visible and obvious components of the text, whereas latent content and involves an interpretation of the underlying meaning of the text [25].

Our analysis involved the following steps: 1) Reading through transcripts several times to obtain a sense of the whole; 2) Extracting text relating to difficulties in ACP uptake and converting the text into meaning units; 3) Summarizing meaning units into condensed meaning units labelled with codes; 4) Comparing and contrasting the codes and sorting them into tentative subcategories and categories; 5) Revising the tentative categories and subcategories into more definitive ones. Quotes were selected through critical discussion between authors. The first author translated the quotes to English while co-authors were responsible for proofreading.

### Trustworthiness

Dependability (stability of findings over time) was achieved through stepwise replication, where three authors analyzed the transcripts separately [26]. The tentative categories and subcategories were then critically discussed and revised. Additionally, the first author adopted the code-recode strategy to code the data twice, giving a 1-month gestation period between codings. Differences between two codings were further discussed with the other two authors, in order to improve presentation of informants’ narratives.

Credibility (truthfulness of research findings and correct interpretation of informants’ original views) was established through peer debriefing [26]. The first author sought comments from experts in geriatric and palliative medicine to improve the credibility of the findings.

## Results

Fifty-one patients and family caregivers initially agreed to participate. Twenty-one of them dropped out on the account of, for example, tiredness and emotional distress. The remaining 17 patients and 13 family members participated in three focus groups (two groups of three patients and one group of two patients) and 22 individual interviews (nine patients and 13 family caregivers). Table 1 summarizes their sociodemographic characteristics.

**Table 1:** Profile of informants (*n* = 30)

Characteristics	Patient (*n* = 17)	Family caregiver (*n* = 13)
	N (%)	N (%)
Gender		
Male	11 (64.7)	3 (25.0)
Female	6 (35.3)	9 (75.0)
Age		
< 60	0 (0.0)	4 (33.3)
60–69	7 (41.2)	4 (33.3)
70–79	7 (41.2)	4 (33.3)
≥ 80	3 (17.6)	0 (0.0)
Marital Status^a^		
Single	0 (0.0)	4 (33.3)
Married	12 (80.0)	7 (58.3)
Divorced	1 (6.7)	0 (0.0)
Widowed	2 (13.3)	1 (8.3)
Religion^a^		
Protestantism	3 (20.0)	3 (25.0)
Taoism	3 (20.0)	1 (8.3)
Buddhism	3 (20.0)	1 (8.3)
Catholicism	0 (0.0)	1 (8.3)
No religious belief	6 (40.0)	6 (50.0)

^a^Items were not total original sample sizes due to missing data

Our qualitative analysis identified four barriers to ACP: 1) limited patients’ participation in autonomous decision making, 2) emotional barriers to discussion, 3) lack of readiness and awareness of early discussion, and 4) unprepared healthcare professionals and healthcare system.

### Limited patients’ participation in autonomous decision making

Instead of indicating own preferences, terminally ill patients tended to leave end-of-life decisions to their families and/or doctors, mainly due to their strong trust in their families and/or doctors and avoidance of decisional conflicts. In addition, family members were reluctant to disclose medical information to the patients or to involve the patients in decision making.

#### Family-centered decision making

A patient was receiving chemotherapy with palliative approach. His treatment decision was not made by himself but his daughter.:

*My daughter didn’t ask my preference … I don’t care what she has discussed with doctors. She knows me very well. (Patient, Male, Age 70–79).*

In addition, family members were reluctant to disclose information regarding medical condition to patients, in order to keep psychological burden away from patients. This can, however, diminish patients’ participation in ACP:

*She will be nervous. This (information disclosure) will only pose a burden on her. Her life should end peacefully. (Family, Female, Age 70–79; Patient, Female, Age ≥ 80).*

#### Doctor-centered decision making

With an absolute trust in doctors, both patients and family members believed that doctors would make the best treatment decisions.

*Personally, I will let the doctor make decisions for me. If he thinks treatment is needed, then I trust him. I always agree with doctors. (Patient, Female, Age 60–69).*

*First, it is because I trust him (doctor). Second, He won’t do something bad for me because ‘Doctoring is parenting’ (a Chinese old saying). If he believes he can save that patient, he must do it. If he thinks (he can’t) … then no need to waste resources. (Family, Female, Age 70–79; Patient, Female, Age ≥ 80).*

Self-perceived incompetence in understanding treatment details was another reason underlying preference for doctor-centered decision making.

*I would follow any instructions from doctors, because I don’t know what (treatments) should be done as the following steps. So just let them decide. (Patient, Female, 60–69).*

#### Avoidance of decisional conflict

More than one treatment option would be discussed in ACP. A patient, however, stated that he could be overwhelmed by multiple options, which in turn discouraged him from discussing:

*No. Sometimes there’s too much information available. If you don’t ask, there’ll only be one direction and you go forward. If you know more, you won’t know which way is better. (Patient, Female, 60–69).*

### Cognitive and emotional barriers to discussion

Several beliefs held by the patients might hinder ACP initiation. Some of them were not interested in conversations since they accept the inevitability of death or prioritize positivity over discussing life issues. Some were unwilling to express their thoughts so as not to burden their families.

#### Prioritizing positivity over life and death

Patients tended to accept the inevitability of death and to stay positive. In order to maintain a peace of mind, they neither actively sought information from doctors nor discussed their medical conditions with doctors. Meanwhile, this prevented them from initiating end-of-life discussion:

*To be honest, absolutely not (for medical information seeking). At our ages, death is inevitable. The most important aspect of one’s life is happiness. I have talked with doctors and have said I don’t care (about prognosis and treatments) … If the doctors say ‘no’ (illness progression), then I will pretend to be fine. (Patient, Female, Age 60–69).*

#### Sense of powerlessness

Patients believed that their destinies, getting terminal illnesses, were predetermined. No matter what they did, they would end up with the same outcome – death. Hence, they consider ACP unnecessary.

*Those who smoke can get lung cancer. Those who don’t smoke can also get lung cancer. How to explain? I rather trust that is my fate. If you are destined for getting it, you can’t avoid it. If not, you won’t get it even you smoke or drink a lot … Just let it go if it cannot be cured (Patient, Male, 70–79).*

#### Avoidance of burdening family

From perspectives of several patients, ACP discussion was complicated. They refused to initiate discussion in order to avoid disturbing daily lives of their families:

*At this stage, you cannot burden your family. My wife and I are of similar age. It’s already very good if she can take care of herself, isn’t it? How can she take care of you (me) as well? Our sons and daughters have to work or go to school. This (talking with family) will only bother them. (Patient, Male, 70–79).*

#### Reluctance to express inner feelings

A family member mentioned that her husband (seriously ill patient) was unwilling to communicate his thought. In her opinion, low education and male gender were the underlying reasons:

*The problem is … you know I am 70 years old. Our generation have poor communication skills. Nowadays people have received education. At least (they) have some basic knowledge of communication and know how to counsel. Our generation don’t have (such knowledge) … To make things worse, he is a male, who is difficult to communicate with, or there is almost no communication. He knows he has a serious illness, but he seldom talks about what he wants, or anything else. (Family, Female, Age 70–79; Patient, Male, Age 70–79).*

### Lack of readiness for and awareness of early discussion

Terminally ill patients might not be ready for communication immediately after acknowledging their diagnosis. In addition, the patients and their family members were not aware of the choice of early discussion as well as its importance. These factors contribute to a delay in ACP initiation.

#### Lack of readiness for early discussion

A family caregiver mentioned that patients might not be ready for discussion immediately after acknowledging their diagnosis and prognosis. Time was needed for patients to accept and overcome their misfortune:

*One and a half year has passed since diagnosis. Actually, just after knowing this news, I have tried to ask him what he was thinking about, what he wanted to do, and which treatment approach he preferred. However, it may be impossible for patients to immediately understand why their conditions could only keep worsening. It might take at least a few months to understand...Of course, the elderly usually tend not to talk about this (death issues). (Family, Female, Age 30–39; Patient, Male, Age ≥ 80).*

#### Lack of awareness of early discussion

A patient believed that she could not participate in ACP discussion, because she would not be able to do so at the end-of-life stage:

*At that moment (end of life) I am already...how can I make a decision? I will leave it to my family for sure. (Patient, Female, 60–69).*

A family member also had not even considered initiating discussion with her loved one:

*We have not talked about it (end-of-life decision making), because health care in Hong Kong is excellent. My father’s condition remains well, so I didn’t have any concern before. But, after this interview, I think I should better consider it. (Family, Female, Age 30–39; Patient, Male, Age ≥ 80).*

### Unprepared healthcare professionals and healthcare system

From perspectives of healthcare users, healthcare professionals were reluctant to and were unable to address medical, informational and emotional needs of the patients and their family members. Furthermore, factors hindering ACP may extend to health system level.

#### Reluctance to withhold futile life-sustaining treatment

Although a patient had expressed reluctance to receive futile life-sustaining treatments, an oncologist still kept persuading him to receive more treatments:

*The consultant told me if I received the treatment then my life could be extended for 2 months. If not, my life would be shortened for 2 months … At that time, I didn’t want to receive (the treatment). But eventually I received the treatment for three times. Later, he said I could continue to undergo the treatment. The more treatments I received, the more sluggish I was. I asked whether I could quit. The consultant questioned me, “Really?” … And in the next consultation, the doctor told me, I could keep receiving treatment in view of my condition … He asked “How about getting the treatment again?” (Patient, Male, Age 60–69).*

#### Unclear explanations about diagnosis, prognosis and treatment

Both patients and families stated that doctors tended to give unclear responses or explanations about medical conditions at early stage. A family caregiver felt helpless when she knew her loved one had a serious illness. She hoped doctors and nurses could explain more to address her needs on information:

*For some patients or their families, the news came like a thunderbolt out of a clear sky. At that moment I was helpless. At the beginning, which means the diagnosis stage, I hope doctors or nurses can explain the medical condition more to patients or their families. (Family, Female, Age 30–39; Patient, Male, Age ≥ 80).*

Meanwhile, our participants interpreted this phenomenon in different ways:

*It depends. Not every doctor explained so clearly. Maybe they did not have time … they had a lot of work. So, they could not answer so many questions (Family, Female, Age 60–69; Patient, Female, Age ≥ 80).*

*They are used to talking like that (giving vague explanation). I have seen a lot of doctors doing that. (Patient, Female, Age 60–69).*

*My mum’s condition keeps worsening. I told the doctor and he did not give me clear responses, probably because he could not help me. (Family, Female, Age 70–79; Patient, Female, Age ≥ 80).*

#### Lack of communication skills and empathy

They also commented that doctors sometimes communicated in an unfriendly manner. This could hamper doctor-patient relationship and hinder effective discussion:

*A doctor yelled “if you don’t receive the treatment you will die” (in an oncology inpatient setting). From the perspective of patients, such words will make us feel down. So I think doctors, I don’t know the reasons, but I think as a professional, the best way to communicate is not to say those words to a person in need … Sometimes their words are discouraging. (Patient, Female, 60–69).*

#### Time constraints

Many participants cited the tight schedule of healthcare professionals as a barrier to discussion:

*I want to know the diagnosis and prognosis. I want to know what will happen if the condition keeps worsening. But doctors (in acute setting) were really too busy. He (doctor) talked with me in the corridor. That’s depressing. (Family, Female, Age 30–39; Patient, Male, Age 80–89).*

#### Lack of care continuity

Under the healthcare system of Hong Kong, patients usually are seen by different doctors across visits in the same clinical settings. They could hardly develop a long-term relationship and have continual communication with the same doctors. This might also prevent continual ACP discussion:

*Consultations are delivered by different doctors. It is not the same person every time. (Patient, Female, 60–69).*

## Discussion

In this qualitative study of 17 seriously ill patients and 13 family members in a Palliative Day Care Centre of Hong Kong, we identify four barriers to ACP uptake. The barriers are discussed one by one as follow. Given that research on Chinese patients reaching the end of life stage undergoing care in hospitals is sparse, our study can fill the research gap.

### Limited patients’ participation in autonomous decision making

While the locus of the western model of medical decision making is the patient, however, this study demonstrates that family members are primary decision makers for Chinese patients. Under Confucian tradition, the Chinese families often function as units in medical decision making [27]. At the same time, Chinese family caregivers are unlikely to disclose diagnosis and prognosis to end-of-life patients. They believe that patients’ awareness of poor prognosis can impair patients’ well-being [28]. The family caregivers may even request doctors to withhold the truth from patients [29]. Information inadequacy can lead to inaccurate expectations in patients and hinder ACP.

In our study, majority of patients and a few family caregivers also preferred doctor-centered decision making, which is attributed to a full trust in doctors. Culturally, older Chinese perceive doctors’ opinions as absolute [30], in the sense that doctors should have ‘final words’ in clinical routine [31, 32]. Furthermore, these patients and family caregivers considered themselves as laymen who cannot make the ‘best’ medical decisions. They tended to leave treatment decisions to discretions of doctors [33]. As a result, both patients and family members were less likely to participate in ongoing ACP process.

### Cognitive and emotional barriers to discussion

Consistent with a Canadian health care professional survey [19], avoidance of death anxiety discouraged our palliative patients from discussion. Additionally, under collectivism in Chinese society, patients may perceive themselves as a burden to their families. The Chinese patients tend to prioritize family interests over self-interests [34]. Moreover, collectivism may induce patients’ unwillingness to express their own wish and preference [35], which further keeps patients from decision making. As a result, end-of-life treatments could be hardly aligned with patients’ individual preferences and values.

### Lack of readiness and awareness of early discussion

This study reaffirms that ACP is not widely recognized by the public of Hong Kong. Surprisingly, a family caregiver had not even considered initiating discussion, due to a lack of awareness of impending death. A patient also did not recognize the opportunity for end-of-life decision making prior to mental incapability. These findings clearly indicate limited awareness of early end-of-life discussion and decision making. Similarly, a local telephone survey reveals that less than 10% of Hong Kong general population had heard of and understood what AD was [36]. Poor knowledge of seriously ill patients and their family caregivers can hinder the ACP implementation and AD documentation.

### Unprepared healthcare professionals and healthcare system

From perspectives of healthcare professionals, “difficulty in accepting a poor prognosis” (emotional aspect) and “difficulty in understanding the limitations and complications of life-sustaining treatments” (informational aspect) among patients and family members are the most significant barriers to goals of care discussion [19, 21, 37]. On the other hand, patients and family members in the present study perceived emotional and informational support from healthcare professionals as insufficient. Healthcare professionals may not know what to say, how to address fear and other emotions, and how to show empathy appropriately [38]. A Hong Kong local survey shows that healthcare workers have a low competence required to cope with emotional and existential challenges from death work, including palliative care, advance care planning discussion and bereavement counselling [7]. They may show discomfort in confronting failure when they cannot cure patients. This is particularly common for prognostication of patients with non-malignant terminal illnesses.

Last but not least, this study reveals that factors hindering ACP may extend to the entire health system. As patients’ preferences may change over time, ACP has to be ongoing and require multiple sessions to complete. However, our patients often saw different doctors in each consultation. Hence, they were unlikely to develop a continuous relationship with the same doctors. Given the lack of ACP documentation revealed in the clinical record review, patient care was fragmented. This may in turn impede implementation of ACP [39]. Aggravated by a short contact time with healthcare professionals especially doctors, ongoing ACP was further unlikely to be initiated [40].

### Study limitations and further study

Our qualitative findings were generated from experience of patients receiving palliative day care services and their family caregivers. The findings may not be generalizable to those receiving other types of care including intensive care units. Nevertheless, the richness of individual experiences and views on both palliative and curative treatments can provide a deep insight into treatment decision making and discussion. Besides, referral from the nurses working in the centre might lead to selection bias. Further studies should recruit patients of heterogeneous characteristics in different settings in order to investigate if the factors hindering ACP uptake identified in this study apply to other palliative as well as non-palliative settings.

## Conclusions

This study identifies factors hindering ACP uptake among seriously ill patients in Hong Kong. From unique perspectives of health service users, the barriers could be related to 1) limited patients’ participation in autonomous decision making, 2) Cognitive and emotional barriers to discussion, 3) lack of readiness and awareness of early discussion, and 4) unprepared healthcare professionals and healthcare system. These findings improve our understanding of patients’ and families’s perspectives and indicate opportunities to facilitate ACP uptake through a series of quality improvement initiatives. To raise capacity among healthcare and social workers, role play and drama centering on commonly occurring end-stage disease scenarios in hospitals may help illustrate different care options and ways to initiate discussion. Another initiative is to run small groups involving end-of-life patients and their family members separately or together, to introduce the topic in a non-threatening way. This may enhance their awareness and readiness of early discussions. An important point is providing emotional support to patients and families during such discussions. Our findings may inform further interventional studies on strengthening ACP and end-of-life communication in a tailored approach for Hong Kong or Chinese population.

With earlier and effective ACP uptake, it is believed that seriously ill patients can make informed decisions and receive care aligned with their individual preferences, leading to a higher quality of life and death.

## Supplementary information

**Additional file 1.** Appendix A – Interview guide for patients.

**Additional file 2.** Appendix B – Interview guide for family caregiver.

## Data Availability

The dataset analyzed in this study is available from the corresponding author on reasonable request.

## Abbreviations

ACP: Advance care planning
AD: Advance directive
