# Supplementary material for: Barriers to advance care planning: a qualitative study of seriously ill Chinese patients and their families
Source: BMC Palliat Care. 2020 Jun 8;19:80. doi: 10.1186/s12904-020-00587-0 (PMC7282137; doi:10.1186/s12904-020-00587-0)
Supplement: Supplementary file 1 — Additional file 1. Appendix A – Interview guide for patients. [file 12904_2020_587_MOESM1_ESM.docx]

**Appendix A – Interview guide for patients**

1. Have you talked to others about your ideas, wishes or other things that are important to you? (e.g. treatment direction) Who do you wish to talk to? Why?
2. Are you eager to know more about your medical condition? (Diagnosis, prognosis, treatment and care plan, etc.) Why?
3. Have you actively asked for information regarding your medical condition? (Diagnosis, prognosis, treatment and care plan, etc.) Who did you ask?
4. Are you satisfied with doctor's explanation of your medical condition? (Diagnosis, prognosis, treatment and care plan, etc.) Why?
5. If your condition worsened, would you still want to know more about your medical condition? (Diagnosis, prognosis, treatment and care plan, etc.) Why?
6. Do you want to make the critical medical decisions on your life (e.g. life sustaining treatment), or leave the decisions to your doctor or family members? Why?
7. Do you think your family members understand your will? Why?
8. Do you think your family members should have a say on your treatment decisions? Why?
