# Supplementary material for: Barriers to advance care planning: a qualitative study of seriously ill Chinese patients and their families
Source: BMC Palliat Care. 2020 Jun 8;19:80. doi: 10.1186/s12904-020-00587-0 (PMC7282137; doi:10.1186/s12904-020-00587-0)
Supplement: Supplementary file 2 — Additional file 2. Appendix B – Interview guide for family caregiver. [file 12904_2020_587_MOESM2_ESM.docx]

**Appendix B – Interview guide for family caregiver**

1. Has the patient talked to others about his/her ideas, wishes or other things that are important to him/her? (e.g. treatment direction) Who do you wish to talk to? Why?
2. Are you eager to know more about patient’s medical condition? (Diagnosis, prognosis, treatment and care plan, etc.) Why?
3. Have you actively asked for information regarding patient’s medical condition? (Diagnosis, prognosis, treatment and care plan, etc.) Who did you ask?
4. Are you satisfied with doctor's explanation of patient’s medical condition? (Diagnosis, prognosis, treatment and care plan, etc.) Why?
5. If patient’s condition worsened, would you still want to know more about his/her medical condition? (Diagnosis, prognosis, treatment and care plan, etc.) Why?
6. Do you think you understand patient’s treatment preference? (e.g. life sustaining treatment) Why?
7. Do you think you should have a say on patient’s treatment decisions? Why?
8. Do you prefer leaving the critical medical decisions to doctor?
